# Failure to account for practice effects leads to clinical misinterpretation of cognitive outcome following carotid endarterectomy

**DOI:** 10.14814/phy2.13264

**Published:** 2017-06-14

**Authors:** Christopher J. Marley, Andrew Sinnott, Judith E. Hall, Gareth Morris‐Stiff, Paul V. Woodsford, Michael H. Lewis, Damian M. Bailey

**Affiliations:** ^1^ Neurovascular Research LaboratoryFaculty of Life Sciences and EducationUniversity of South WalesWalesUnited Kingdom; ^2^ Department of Anaesthetics and Intensive Care MedicineCardiff UniversityWalesUnited Kingdom; ^3^ Department of HPB SurgeryDigestive Disease InstituteCleveland Clinic FoundationClevelandOhio; ^4^ Department of SurgeryRoyal Glamorgan HospitalWalesUnited Kingdom; ^5^ Faculty of MedicineReichwald Health Sciences CentreUniversity of British Columbia‐OkanaganKelownaBritish ColumbiaCanada

**Keywords:** Carotid endarterectomy, cerebral ischemia–reperfusion, cognitive function, postoperative, practice effects

## Abstract

Carotid endarterectomy (CEA) is a surgical procedure to remove stenotic atherosclerotic plaque from the origin of the carotid artery to reduce the risk of major stroke. Its impact on postoperative cognitive function (POCF) remains controversial; complicated, in part, by a traditional failure to account for practice effects incurred during consecutive psychometric testing. To address this for the first time, we performed psychometric testing (learning and memory, working memory, attention and information processing, and visuomotor coordination) in 15 male patients aged 68 ± 8 years with symptomatic carotid stenosis the day before and 24 h following elective CEA (two consecutive tests, 48 h apart). Multiple baselining was also performed in a separate cohort of 13 educationally, anthropometrically and age‐matched controls (63 ± 9 years) not undergoing revascularization at identical time points with additional measures performed over a further 96 h (four consecutive tests, each 48 h apart). A single consecutive test in the control group resulted in progressive improvements in learning and memory, working memory, and attention and information (*P *<* *0.05 vs. Test 1), with three tests required before cognitive performance stabilized. Following correction for practice effects in the patient group, CEA was associated with a deterioration rather than an improvement in learning and memory as originally observed (*P *<* *0.05). These findings highlight the potential for the clinical misinterpretation of POCF unless practice effects are taken into account and provide practical recommendations for implementation within the clinical setting.

## Introduction

Carotid endarterectomy (CEA) is a surgical procedure to remove stenotic atherosclerotic plaque from the origin of the carotid artery to reduce the risk of major stroke (North American Symptomatic Carotid Endarterectomy Trial 1991). While it has the capacity to improve postoperative cognitive function (POCF) through restoration of cerebral perfusion and oxygenation, complications associated with intraoperative embolization and hemodynamic impairments subsequent to obligatory surgical ischemia–reperfusion may contribute toward cognitive decline (Bailey et al. 2007). To what extent these changes collectively impact on a patient's overall cognitive outcome in the short‐ or long‐term remains unclear, with studies reporting either an improvement, no change, or indeed, deterioration (De Rango et al. 2008; Paraskevas et al. 2014; Plessers et al. 2014). Given the need for consecutive testing, a consistent failure to adequately account for practice effects has been suggested as arguably one of the most important experimental limitations that has contributed toward the controversy (Irvine et al. 1998; De Rango et al. 2008; Paraskevas et al. 2014; Plessers et al. 2014, 2015).

Practice effects refer to the familiarization of previous cognitive testing procedures characterized by an artifactual improvement in performance (McCaffrey and Westervelt 1995). These effects are most pronounced following the short‐term reassessment of cognition (Theisen et al. 1998) (e.g., the first few days following CEA), but may also extend to longer retest intervals (Plessers et al. 2015). This is of concern, as it can complicate clinical interpretation following CEA and subsequently result in misdiagnosis. Therefore, methods capable of minimizing practice effects are warranted.

To date, a variety of methods have been adopted in an attempt to control for practice effects including alternative versions of the tests, modified reliable change indices (i.e., statistical methods), and control groups. However, each approach has its limitations. Alternative versions of the tests have been utilized with some success (Benedict and Zgaljardic 1998), but patients are still thought to become “test wise,” thereby confounding performance (Irvine et al. 1998; Paraskevas et al. 2014; Plessers et al. 2014, 2015). Modified reliable change indices can also be calculated by dividing the individual's test–retest difference score by the standard error of that difference (Jacobson and Truax 1991; Chelune et al. 1993). However, this method requires test–retest data from appropriately matched control groups, which are difficult to obtain within the clinical setting (Collie et al. 2002).

Multiple baselining represents an alternative approach to minimize practice effects. This refers to the repeated administration of cognitive tests, with the aim of producing a more stable baseline, since practice effects would have already occurred (McCaffrey and Westervelt 1995). Traditionally, two baseline measurements are recorded, with the second assessment used as the “true” baseline (Collie et al. 2002). However, the precise number of repeated baselines necessary to achieve habituation (i.e., for cognitive performance to stabilize) remains to be established.

In light of these findings, the present study sought to quantify practice effects incurred during two consecutive tests in a healthy control group not undergoing revascularization, and further establish the number of multiple baselines required for cognitive performance to stabilize. This information was subsequently applied to a patient group in an attempt to “normalize” test scores to provide clearer insight into the corresponding impact of CEA on POCF within 24 h of surgery, a clinically meaningful endpoint traditionally reported in the literature that provides early insight into the success of the surgical intervention. We hypothesized that normalization would result in a general deterioration in POCF that would otherwise be misinterpreted for an improvement had practice effects not been taken into account, and that more than two consecutive tests would be required for habituation to occur.

## Methods

### Ethics

The study was approved by the local Ethics Committee (#09/WSE03/47). All procedures were carried out in accordance with the Declaration of Helsinki of the World Medical Association with oral and written informed consent obtained from all participants.

### Patient study

Fifteen consecutive, right‐handed male patients scheduled for elective unilateral CEA were recruited. All patients presented with a history of amaurosis fugax (*n *=* *7) and/or transient ischemic attacks (*n *=* *10) with 85 ± 8% stenosis at the bifurcation of the internal carotid artery according to established criteria (Trial North American Symptomatic Carotid Endarterectomy C 1991). Patients were instrumented for continual recording of intra‐arterial blood pressure and heart rate via electrocardiography (leads II and V5) before placement of the anesthetic block. All patients were submitted to the block of deep and superficial cervical plexus, in the supine position with the head facing away from the side to be blocked. Patients received fentanyl (20 mg preop local, 10 mg preincision, 5 mg during the procedure) and midazolam (3 mg preop local, 0.5 mg during the procedure) with local anesthesia achieved via lidocaine (2%, 10 mL) and ropivacaine (0.75%, 15 mL).

All surgeries were performed under local anesthesia by the same consultant surgeon (MHL).

### Control study

Following completion of the patient study, 15 healthy asymptomatic males not scheduled for carotid surgery were recruited as control comparators. Participants were prospectively matched for age, body mass index, education, hand dominance, and lifetime physical activity levels defined as sedentary with no formal recreational activity outside of everyday living (Bailey et al. 2013).

### Design

All participants completed a standard battery of psychometric tests that were counterbalanced and performed at an identical time of day by the same trained investigator. In the patient study, cognitive function was assessed the day before and exactly 24 h following CEA (two repeat tests that were 48 h apart). In the control study, cognitive function was assessed at identical time points including an additional two tests (four repeat tests each separated by 48 h).

### Cognitive function

The psychometric tests included the core tests according to the recommendations of the statement of consensus on the assessment of neurobehavioral outcomes after cardiac surgery (Lloyd et al. 2004). Each test was divided further into the following cognitive domains: *learning and memory* (Rey Auditory Verbal Learning Tests A [RAVLT‐A; sum of A1‐A5] and B [RAVLT‐B; A6 minus A5]), *working memory* (Repetition of Digits Backwards [RDB; longest span]; Trail Making Test B [TMT‐B]), *attention and information processing* (Repetition of Digits Forwards [RDF; longest span]; Trail Making Test A [TMT‐A]; Digit Symbol Substitution Test [DSST]), and *visuomotor coordination* (Grooved Pegboard Dexterity Test using both the dominant [GPD] and nondominant [GPND] hand). Higher scores in the RAVLT‐A/B (Rey 1941), RDB and RDF (Wechsler 1945), as well as the DSST (Weschler 1989) tests are indicative of superior performance. Conversely, lower scores in the TMT‐A/B (Weschler 1989) as well as the GPD and GPND (Trites 1977) (Lafayette Instruments, Loughborough, Leicestershire, UK) are indicative of superior performance (i.e., the task was completed quicker).

### Practice effects (control study)

The practice effect for each respective pscychometric test was calculated during consecutive testing in the control study given by:

(1) $$\text{Practice effect}=\left(\frac{\text{Test 2 ‐ Test 1}}{\text{Test 1}}\right)\times 100(\%)$$

An individual correction factor was calculated for each pscychometric test given by:

(2) $$\text{Correction factor}=1-\left(\frac{\text{Practice effect in}\%}{100}\right)$$

### Correction of postoperative cognitive function scores (patient study)

These correction factors were retrospectively multiplied against each of the patient's postoperative cognitive function scores in order to normalize for practice effects and yield the patient's (true) scores. The corresponding implications for clinical interpretation (corrected vs. uncorrected) were determined by comparing differences in cognitive function outcome given by:

(3) $$\text{Difference}=\left(\frac{\text{Postop (corrected or uncorrected) ‐ Preop}}{\text{Preop}}\right)\times 100\left(\%\right)$$

A worked example based on actual data for the purposes of clarification is outlined in the Results.

### Statistical analysis

#### Inferential statistics

Data were analyzed with the Statistics Package for Social Scientists (IBM SPSS Statistics version 22.0). Following confirmation of distribution normality (Shapiro Wilk *W* tests), independent samples *t*‐tests were used to compare the baseline characteristics of the patients and controls. Changes in POCF following CEA and as a function of practice effects in the control group were analyzed using paired samples *t*‐tests. A one‐way repeated measures analysis of variance and Bonferonni‐corrected paired samples *t*‐tests were used to determine the effects of consecutive testing in the controls. Power and effect size for each reported outcome were retrospectively calculated using Cohen's equation (Cohen 1992) and reported as a *d* value. Significance was established at *P *<* *0.05 for all two‐tailed tests and data expressed as mean ± SD.

#### Critical difference

Data obtained from the control study were also used to calculate the critical difference (CD) for each of the cognitive parameters assessed. For the first time, this allowed us to determine to what extent the observed changes in cognitive function were clinically significant, that is, exceeded the “background noise” associated with normal biological variation (Fraser and Fogarty 1989; Bailey et al. 2016). This was calculated as:

$$\text{CD}=k\sqrt{{\text{CV}}_{A}^{2}+{\text{CV}}_{B}^{2}}$$

where *k* is a constant equal to 2.77 at *P *<* *0.05, CV_A_ is the coefficient of analytical variation (assumed to be 0 given the manual nature of the cognitive tests employed, i.e., no electronic component or calibration required), and CV_B_ is the coefficient of biological variation (calculated from repeated measures within the control study).

## Results

### Baseline characteristics

Table 1 confirms that controls and patients were well‐matched with the inevitable exception of cardiocerebrovascular risk profile and medication. Aspects of cognitive function (learning and memory, visuomotor coordination) were impaired in the patients, confirmed by lower RAVLT‐A/RAVLT‐B and higher GPND scores (*P *<* *0.05).

**Table 1:** Baseline characteristics

Measurement	Controls (*n *=* *13)	Patients (*n *=* *15)
Demographics		
Age (years)	63 ± 9	68 ± 8
Body mass index (kg/m^2^)	27 ± 5	30 ± 4
Education (years)	13 ± 2	14 ± 4
Medication		
Aspirin (*n*/%)	/	12 (80)
Warfarin (*n*/%)	/	5 (33)
Clopidogrel (*n*/%)	/	4 (27)
Beta‐blockers (*n*/%)	/	5 (33)
ACE inhibitors (*n*/%)	/	6 (40)
Statins (*n*/%)	/	7 (47)
Calcium channel antagonists (*n*/%)	/	4 (27)
Cognitive function		
Learning and memory		
RAVLT‐A (*n*)	46 ± 13	37 ± 9^a^
RAVLT‐B (*n*)	−2 ± 2	−4 ± 2^a^
Working memory		
RDB (*n*)	6 ± 2	5 ± 2
TMT‐B (sec)	95 ± 55	105 ± 44
Attention and information		
RDF (*n*)	7 ± 2	8 ± 3
TMT‐A (sec)	39 ± 14	44 ± 12
DSST (*n*)	50 ± 15	42 ± 9
Visuomotor coordination		
GPD (sec)	81 ± 22	99 ± 27
GPND (sec)	84 ± 20	108 ± 28^a^

Values are mean ± SD. RAVLT‐A/B, Rey Auditory Verbal Learning Test parts A and B; RDB, Repetition of Digits Backwards; TMT‐B, Trail Making Test part B; RDF, Repetition of Digits Forwards; TMT‐A, Trail Making Test part A; DSST, Digit Symbol Substitution Test; GPD and GPND, Grooved Pegboard Test using both dominant and nondominant hands; *n*, number correct.

a Different versus controls (*P *<* *0.05).

### Control study

Two participants were excluded from the overall analyses due to loss to follow‐up. In the remaining 13 participants, a single consecutive test was shown to improve RAVLT‐A (*d = *0.81), TMT‐A (*d = *−0.39), and DSST (*d = *0.20, *P *<* *0.05 vs. Test 1), whereas the remaining parameters remained unchanged (Table 2). Three consecutive tests were required for RAVLT‐A, TMT‐A, TMT‐B, and DSST (aspects of learning, working memory, and attention and information) to stabilize before plateauing whereby no further improvements were observed (Table 2). All practice effects were within the calculated CDs that ranged between 13% and 97%.

**Table 2:** Cognitive function during consecutive testing in controls

Cognitive domain	Test	**Test 1**	**Test 2**	Test 3	Test 4	Practice effect (%)	CD (%)	POCF correction factor
Learning and memory	RAVLT‐A (*n*)	**46 ± 13**	**61 ± 13** a	65 ± 10^a^	67 ± 10	42 ± 14	44	0.58
	RAVLT‐B (*n*)	**−2 ± 2**	**−1 ± 2**	−1 ± 1	−1 ± 2	35 ± 0	97	0.10
Working memory	RDB (*n*)	**6 ± 2**	**5 ± 1**	6 ± 2	6 ± 1	−9 ± 21	20	0.91
	TMT‐B (sec)	**95 ± 55**	**86 ± 56**	81 ± 38^a^	75 ± 35	−10 ± 22	27	1.10
Attention and information	RDF (*n*)	**7 ± 2**	**7 ± 1**	7 ± 1	7 ± 2	−5 ± 27	13	0.95
	TMT‐A (sec)	**39 ± 14**	**32 ± 7** a	35 ± 11	38 ± 15	−13 ± 20	23	1.13
	DSST (*n*)	**50 ± 15**	**55 ± 16** a	58 ± 14	58 ± 16	9 ± 10	19	0.91
Visuomotor coordination	GPD (sec)	**81 ± 22**	**76 ± 18**	70 ± 15	67 ± 11	−6 ± 13	23	1.06
	GPND (sec)	**84 ± 20**	**81 ± 20**	74 ± 13	73 ± 17	−4 ± 9	20	1.04

Values are mean ± SD. Practice effect calculated as the improvement from Test 1 to Test 2 [Test 2 − Test 1/Test 1 (×100), values represented in bold]; CD, critical difference. POCF (postoperative cognitive function) correction factor (to be multiplied against the respective postoperative score during the patient study) calculated as 1 − (Practice Effect in %/100). RAVLT‐A/B, Rey Auditory Verbal Learning Test parts A and B; RDB, Repetition of Digits Backwards; TMT‐B, Trail Making Test part B; RDF, Repetition of Digits Forwards; TMT‐A, Trail Making Test part A; DSST, Digit Symbol Substitution Test; GPD and GPND, Grooved Pegboard Test using dominant and nondominant hands; *n*, number correct.

a Different versus preceding test (*P *<* *0.05).

### Patient study

**Figure 1:**
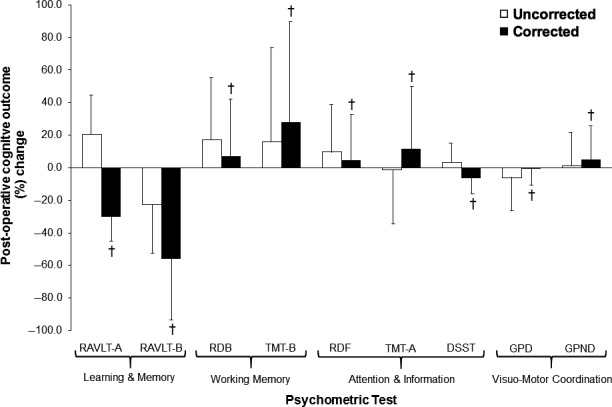
Importance of correcting for practice effects during the clinical interpretation of postoperative cognitive outcome in patients undergoing carotid endarterectomy (CEA). Values are mean ± SD. Percent (%) change calculated as postop (corrected or uncorrected) − preop/preop × 100. RAVLT‐A/B, Rey Auditory Verbal Learning Test parts A and B; RDB, Repetition of Digits Backwards; TMT‐B, Trail Making Test part B; RDF, Repetition of Digits Forwards; TMT‐A, Trail Making Test part A; DSST, Digit Symbol Substitution Test; GPD and GPND, Grooved Pegboard Test using both dominant and nondominant hands. ^†^Different versus uncorrected (*P *<* *0.05).

Pre‐ and intraoperative sedation and anesthesia was identical for all patients. Furthermore, intraoperative shunting was not required and surgery was successful without complication. CEA was associated with an improvement in (observed, uncorrected scores) RAVLT‐A (*d = *0.51) and deterioration in RAVLT‐B (*d = *−0.25, *P *<* *0.05, Table 3). Following mathematical correction for practice effects identified in the control study (see later for worked example), RAVLT‐A to the contrary translated into a deterioration in performance (*d = *−0.88), while RAVLT‐B became further impaired (*d = *−0.67, Table 3 and Fig. 1). Corrected scores (Table 3, highlighted in black) and corresponding percent change in performance (Fig. 1, based on eq. (3)) in the remaining psychometric tests following CEA were consistently more impaired when compared to the uncorrected postoperative scores (*P *<* *0.05).

**Table 3:** Impact of surgery on cognitive function

Cognitive domain	Test	Pre‐CEA (baseline)	Post‐CEA (uncorrected)	**Post‐CEA (corrected)**	Clinical interpretation
Learning and memory	RAVLT‐A (*n*)	37 ± 9	44 ± 12^a^	**26 ± 7** a, b	Improvement → Impairment
	RAVLT‐B (*n*)	−4 ± 2	−5 ± 2^a^	**−6 ± 2** a, b	Impairment → Further impairment
Working memory	RDB (*n*)	5 ± 2	6 ± 2	**5 ± 2** b	No change
	TMT‐B (sec)	105 ± 44	118 ± 69	**129 ± 76** b	No change
Attention and information	RDF (*n*)	8 ± 3	8 ± 2	**8 ± 2**	No change
	TMT‐A (sec)	44 ± 12	42 ± 14	**47 ± 16** b	No change
	DSST (*n*)	42 ± 9	44 ± 11	**40 ± 10** b	No change
Visuomotor coordination	GPD (sec)	99 ± 27	90 ± 22	**96 ± 23** b	No change
	GPND (sec)	108 ± 28	107 ± 24	**111 ± 25** b	No change

Values are mean ± SD. RAVLT‐A/B, Rey auditory verbal learning test parts A and B; RDB, Repetition of digits backwards; TMT‐B, trail making test part B; RDF, Repetition of digits forwards; TMT‐A, trail making test part A; DSST, Digit Symbol Substitution Test; GPD and GPND, grooved pegboard test using both dominant and nondominant hands; *n*, number correct. Corrected data (values represented in bold) corrected for practice effects.

a Different versus pre‐CEA (*P *<* *0.05).

b Different versus uncorrected (*P *<* *0.05).

### Normalization of postoperative cognitive function for practice effects: worked example

#### Control study

##### Practice effects

Taking performance on the RAVLT‐A based on consecutive testing in the control study and corresponding calculation of the practice effect (eq. (1)) yields:

Calculated practice effect (i.e., improvement) for RAVLT‐A = 42 ± 14% (Table 2).

##### Correction factor

A corresponding correction factor was calculated for each test given by:

$$\begin{array}{cc} \text{Correction factor} & =1-\left(\frac{\text{Practice effect in}\%}{100}\right)\\ & =0.58 \end{array}$$

#### Patient study

Patient SM‐01 who had undergone an elective carotid endarterectomy (CEA) presented with the following RAVLT‐A scores:

Preoperative score: 34

Postoperative (raw, uncorrected) score: 50

##### Traditional interpretation

By ignoring practice effects, this would be interpreted as:

$$\begin{array}{cc} \text{Difference} & =\left(\frac{\text{Postop ‐ Preop}}{\text{Preop}}\right)\times 100\left(\%\right)\\ & =\left(\frac{50-34}{34}\right)\times 100=47\%\;\text{(i.e., improvement)} \end{array}$$

##### Revised interpretation

Preoperative score: 34

Postoperative (corrected) score: 50 × 0.58 = 29

Accounting for practice effects, this would be interpreted as:

$$\begin{array}{cc} \text{Difference} & =\left(\frac{\text{Postop}-\text{Preop}}{\text{Preop}}\right)\times 100\left(\%\right)\\ & =\left(\frac{29-34}{34}\right)\times 100\left(\%\right)=-15\%\;\text{(i.e., impairment)} \end{array}$$

Note that the qualitative and quantitative outcomes following surgery are different; performance on this psychometric test was impaired rather than improved when practice effects are taken into account.

## Discussion

Consistent with our original hypothesis, the present study highlights the extent to which practice effects have the potential to confound the clinical interpretation of POCF in patients undergoing CEA. Indeed, correction for practice through inclusion of a control group translated into a deterioration in learning and memory that would have otherwise been misinterpreted for an improvement with up to three repeat tests required for cognitive performance to stabilize. Collectively, these findings have important clinical implications for patients undergoing CEA.

Following correction for practice effects, all measures of cognitive function were consistently more impaired following CEA. This finding supports similar studies that have investigated the short‐term effects of CEA on cognition (Gaunt et al. 1994; Heyer et al. 2002, 2006, 2013; Mocco et al. 2006; Capoccia et al. 2010; Wasser et al. 2012) and raises the worrying possibility that previous reports that have lacked experimental controls may have underestimated the degree of cognitive decline. Although clearly a life‐saving surgical intervention (North American Symptomatic Carotid Endarterectomy Trial, 1991), CEA is not without risk. Cerebral hypoperfusion during clamp application and subsequent hyperperfusion/scattering of microemboli subsequent to clamp release have the collective potential to adversely impact POCF (Lloyd et al. 2004; De Rango et al. 2008).

Statistical models have been introduced in an attempt to account for the potential confounds associated with practice effects, albeit limited by a reliance on large sample sizes to obtain stable estimates (Ferrer et al. 2004). Multiple baselining may therefore provide an alternative method until such times as a nonhabituating marker of POCF has been developed. The test–retest period employed in the present study (four consecutive days) was specifically designed to define, for the first time, the precise number of repeated baselines required for practice effects to stabilize. We found that cognitive scores for RAVLT‐A, TMT‐A, TMT‐B, and DSST progressively improved over three consecutive tests before plateauing. From a practical perspective, our findings suggest that patients need to complete three practice sessions prior to experimental or clinical data collection before habituation is complete to provide more accurate insight into the short‐term cognitive implications of surgery, a recommendation we consider to be both realistic and feasible to administer in the acute clinical setting. However, it is worth noting that the practice effects observed in the control study were lower than the calculated CDs for each measure, that is, they were within the boundaries of “normal” biological variation, a concept all too often ignored within the clinical literature (Bailey et al. 2016). Nonetheless, these calculations provide useful reference data that can be used to modify data interpretation and/or facilitate prospective power calculations based on what one would consider to be clinically meaningful changes in cognitive outcome measures to optimize the statistical power of future experimental designs.

Learning and memory as assessed by the RAVLT‐A appeared to be the cognitive domain that benefited most from CEA (prior to correction), a finding that is broadly consistent with the literature (De Rango et al. 2008; Plessers et al. 2014). However, this may simply be a reflection of the test's inherent sensitivity to learning given that it increased the most with practice. Indeed, following correction, this improvement was reversed, translating into a deterioration. In contrast, the remainder of the psychometric tests were not statistically different following CEA relative to the preoperative baseline control. It is also important to highlight that not all cognitive tests were subject to practice effects in the control study. No differences were observed in the RAVLT‐B, RDF, RDB, or grooved pegboard tests when repeated over 4 consecutive days in the control group, suggesting that these tests may be more reliable measures for the assessment of short‐term changes in POCF.

A potential limitation of the present study relates to our choice of (healthy) control group since they did not constitute patients suffering with asymptomatic carotid stenoses who chose not to undergo revascularization, arguably considered the ideal comparator (Plessers et al. 2014). This was not feasible in the current study owing to ethical constraints and logistical challenges associated with prospective matching of established cardiocerebrovascular risk factors and best care medication (Collie et al. 2002; Bailey et al. 2006). We employed healthy asymptomatic volunteers as an alternative comparator though great care was taken to match for other potential confounders including age, BMI, education, hand dominance, and physical activity levels. While this likely limited, albeit failed to ablate the subtle differences in cognitive function at baseline (more impaired in patients owing to pathology), there is no published evidence indeed mechanistic basis to suggest that practice effects would have been less pronounced, a concept that warrants future consideration. Finally, to what extent the drugs required for sedation and local anesthesia potentially influenced POCF remains unclear given the obvious ethical constraints (i.e., non‐revascularization control group required), albeit unlikely given that they constitute high clearance drugs that are rapidly and extensively metabolized mainly by cytochrome P450 3A4. Furthermore, all patients received identical pre‐ and intraoperative sedation/anesthesia, thus eliminating potential confounds associated with interpatient variability if indeed residual effects were apparent.

In conclusion, the present findings suggest that CEA adversely affects POCF. From a clinical perspective, we highlight the potential for misinterpreting POCF unless practice effects are taken into account. Until an alternative nonhabituating biomarker of neurocognitive dysfunction is developed, multiple baselining can be employed within the clinical setting to counteract practice effects and provide clearer insight into the short‐term cognitive implications of surgery.

## Conflict of Interest

None declared.
